# Primary productivity connects hilsa fishery in the Bay of Bengal

**DOI:** 10.1038/s41598-020-62616-5

**Published:** 2020-03-27

**Authors:** M. Shahadat Hossain, Subrata Sarker, S. M. Sharifuzzaman, Sayedur Rahman Chowdhury

**Affiliations:** 1 Institute of Marine Sciences, University of Chittagong, Chittagong, 4331 Bangladesh; 2 Department of Oceanography, Shahjalal University of Science and Technology, Sylhet, 3114 Bangladesh

**Keywords:** Ecological modelling, Ecosystem ecology

## Abstract

Tropical hilsa shad (*Tenualosa ilisha*) contributes significantly to the society and economy of Bangladesh, India and Myanmar, but little is known about their habitats across the life cycle and their relationship with environmental drivers. This study describes spatial and temporal variability of productivity in the Bay of Bengal (BoB) relating to hilsa fishery. Decadal data on net primary productivity, nutrients (i.e. nitrate, phosphate and silicate) and zooplankton were collected from Aqua MODIS, world ocean database and COPEPOD respectively with spatial resolution 1°×1°. Moreover, monthly abundance of phytoplankton, hilsa catch and long-term catch dynamics were analyzed to determine the associations between variables. The present study was extended over 3.568 million km^2^ area, of which 0.131–0.213 million km^2^ area characterized as the most productive with net primary production of >2,000 mg C/m^2^/day, 0.373–0.861 million km^2^ area as moderately productive with 500–2,000 mg C/m^2^/day, and 2.517–3.040 million km^2^ area as the least productive with <500 mg C/m^2^/day which were consistent with field verification data. In case of nutrients, the Ganges-Brahmaputra-Meghna (GBM) delta was rich in nitrate and phosphate than that of the Ayeyarwady delta, while silicate concentration persisted high all over the northern BoB including the deltas. A peak abundance of phytoplankton was observed in GBM delta during the months of August-November, when ~80% of total hilsa are harvested in Bangladesh annually. Variations in seasonal productivity linked with nutrients and phytoplankton abundance are important factors for predicting hilsa habitat and their migration patterns in the deltaic regions and shelf waters of BoB. These results can be useful in forecasting potential responses of the hilsa in BoB ecosystem to changing global ocean productivity.

## Introduction

The Bay of Bengal (BoB), which shares many characteristics of the Indian Ocean, is a distinctive system characterized by shallow oceanic arm, deposition of sediment, freshwater plume, seasonal reversal of ocean currents, semidiurnal tides, oxygen-rich surface waters, and abundant biodiversity and fisheries resources^1^. The northern BoB has the widest shallow shelf region, extending more than 100 nautical miles (=185 km), which is 3–4 times wider in Bangladesh than that in the Myanmar, eastern coast of India and a global average of 65 km. Nutrient is the major driver of primary productivity in BoB^2,3^. The coupled climate-oceanographic processes (e.g. atmospheric depressions and tropical cyclone, storm surge, internal wave, eddy pumping, and river inputs) are injecting nutrients to the shallow zone and thereby promoting the primary production in upper layers of coastal waters^4–6^, although productivity of an area or ecosystem is dependent on many other bio-physico-chemical parameters such as light^7,8^.

Phytoplankton is the base of marine food web and thus primary productivity is a key driver of zooplankton and ichthyoplankton dynamics influencing the planktivorous and predatory organisms, mammals, and seabirds^9^. It accounts for ~50% of global primary productivity^10,11^, and is important to ecological, biological and biogeochemical processes in oceans^12,13^. Moreover, marine phytoplankton annually fixes 30–50 billion metric tons of carbon equivalent to 40–50% of the global total^14^. The majority of phytoplankton species require nitrate, phosphate, iron, molybdenum and copper for growth and reproduction, while diatoms have special requirement of silicate for cell structure and metabolism. Thus, changes in nutrient types and concentrations may change phytoplankton species composition and growth rate, and the resultant net primary production^15,16^. Zooplankton and herbivore fishes are directly dependent on phytoplankton for food and thus effect primary production through top-down control^17^. Moreover, productivity (=15–30% of total primary production) is necessary for sustaining the pelagic fish communities^18,19^. Overall, primary productivity is reported to control the abundance, recruitment, migration pattern and yields of pelagic and migratory fisheries^20–22^, which are important sources of human food, protein, employment and livelihoods^17^.

**Figure 1:**
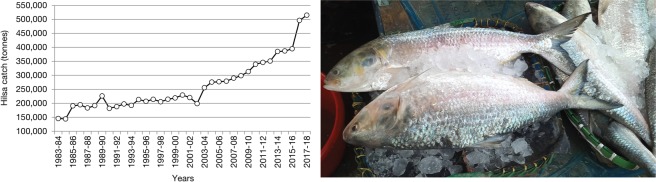
Annual hilsa catch in Bangladesh from 1983 to 2018 (*left*); and few hilsa fishes at a landing center (*right*).

The anadromous hilsa shad (*Tenualosa ilisha*), which feeds on phytoplankton, zooplankton, ichthyoplankton, protozoa, small crustacean and molluscs larvae^23–25^, moves into and out of deltaic ecosystems depending on foraging density and water flow patterns^22,26,27^. Hilsa acquire energy reserves in the offshore waters until reaching maturity, after which they start upstream spawning migration^28^, appear to protect reproductive value^29,30^ with similar behavior in other anadromous fishes, like salmonids and gobiids^31,32^. Hilsa is mainly harvested from coastal and marine waters (72%), and a quarter from freshwater rivers^33^. Bangladesh (76%), Myanmar (15%) and India (4%) contribute 95% of the global hilsa catches; while the remaining 5% shared by Iraq, Iran, Kuwait, Thailand and Pakistan^34,35^. Over the past three decades hilsa production has been increased in Bangladesh with annual harvest reaching 0.5 million tonnes (Fig. 1) that supports livelihoods of 0.5 million fishermen directly, 2.5 million people in the value chain and distribution, and a business valued at US$2 billion^33^. While peak hilsa fishing takes place from July to November, little is known about their spawning, feeding and/or transboundary migration as well as their spatial and temporal distribution in response to primary production. This study aims to map the productivity zones in the Bay of Bengal and explore their relationship with hilsa fishery.

## Materials and Methods

### Study area

**Figure 2:**
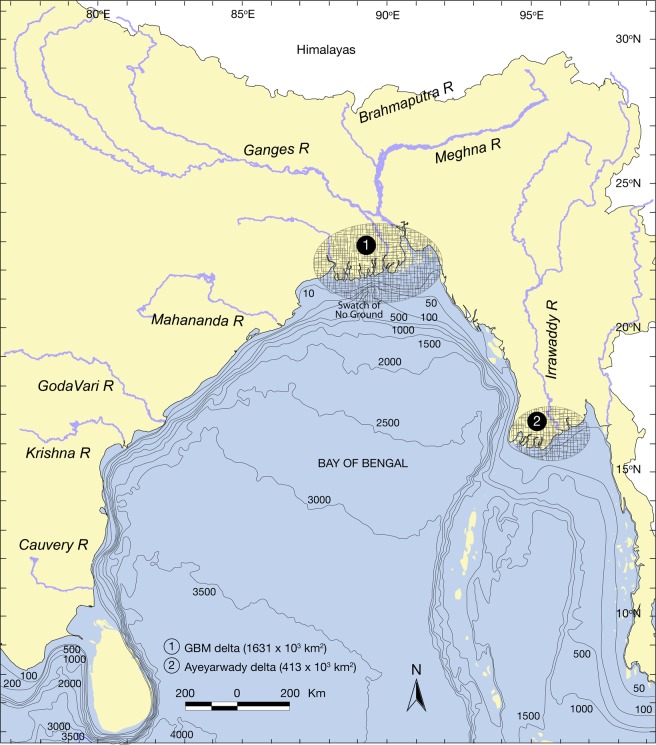
The Bay of Bengal showing Ganges-Brahmaputra-Meghna (GBM) and the Ayeyarwady deltas (bathymetry in meter).

The present study was carried out in the Bay of Bengal covering an area of 3.6 × 10^6^ km^2^ between latitude 5° and 24°N, and longitude 79° and 100°E (Fig. 2) covering marine waters of Sri Lanka, India, Bangladesh, Myanmar, Thailand and Indonesia. Major river systems in the study area were Ganges-Brahmaputra-Meghna (GBM) and Ayeyarwady including their tributaries. The BoB has been governed by southwest monsoon winds from May to October and northeast monsoon winds from November to April^36,37^. Depth of the BoB varies between 10 m in the shelf area of Bangladesh to more than 4500 m as it approaches the Equator^38,39^. The GBM and Ayeyarwady river systems discharge about 1400 km^3^/year freshwater, while precipitation (i.e. precipitation over BoB) contributing 5,900 km^3^/year into the northern BoB^40^, making it fresher as “river in the sea”^41,42^. This freshening signal concentrate within the upper 40 m and spreads southward as a narrow strip (~100 km wide) along the western boundary of the bay and reaches the Indian Ocean after 3–5 months journey^43,44^. Also, freshwater flows southward along the eastern boundary of the basin and gradually mixes with the underlying oceanic layer by vertical exchanges^45–47^. In contrary, the deep canyon in GBM delta (i.e. Swatch of No Ground) has no evidence for sinking freshwater but supports sediment transport to the submarine fan^38^.

### Data and software

Monthly gridded data during 1998–2018 on net primary productivity (NPP) were collected from Aqua MODIS from NOAA (https://coastwatch.pfeg.noaa.gov) in the area between 5°N–24°N and 79°E–100°E corresponding to the Bay of Bengal. Nutrients data (i.e. silicate, nitrate and phosphate) were collected from the world ocean database (https://www.nodc.noaa.gov/) for the same study domain. Zooplankton data were collected from the Coastal & Oceanic Plankton Ecology, Production, & Observation Database (COPEPOD) (https://www.st.nmfs.noaa.gov/copepod/). The NetCDF format data, which were level 3 products (e.g. SeaWiFS, MODIS and MERIS) with spatial resolution 1° latitude × 1° longitude grid^48^ (Kodama *et al*. 2017), were downloaded and geospatially analyzed in ArcGIS software. Marine regions’ data of International Hydrographic Organization (IHO) Sea Areas (version 3) for the Bay of Bengal were collected from http://www.marineregions.org/ and used as base map. The monthly net primary productivity was rated in terms of significance for hilsa habitat modeling (Table 1). The spatial extension module was used for surface interpolation in ArcGIS. All maps and data were transformed into decimal degrees projection. Monthly phytoplankton abundance in the GBM delta was collected from Zafar^49^. Long term hilsa catch data of 1983–2018 was collected from the Department of Fisheries (DoF), Government of Bangladesh and used for the analysis of hilsa catch dynamics. Besides, monthly hilsa catch data were generated through focus group discussions (FGD) and key informant interviews (KII) with experienced fishers, fish traders and distributors in the coastal fishing villages^50^.

**Table 1:** Modeling of net primary productivity.

Productivity rating and score	NPP (mg C/m^2^/day)	Reference
Most productive (3)	>2,000	Akester^4^; Thaw *et al*.^55^
Moderately productive (2)	500–2,000	Perry and Schweigert^60^
Least productive (1)	<500	Thaw *et al*.^55^; Odum^59^

### Data integration

The shape file of study area was converted to grid with cell size of 0.0085 degree, equivalent to 4 km to prepare the mask layer with values 1 inside the mask and 0 outside. The geospatial tabular data of monthly net primary productivity were converted to grids by interpolation, maintaining the same geographic extent and cell size as the mask^51^. In map calculation, individual parameter grid layers multiplying with the mask layer thus resulted in masked grid layers of corresponding months. These parameter grid layers were then reclassified into three classes with <500, 500–2,000 and > 2,000 mg C/m^2^/day as most productive, moderately productive and least productive zones (Table 1) respectively, and then evaluated by adding respective months. The two seasons i.e. southwest monsoon (SWM) and northeast monsoon (NEM) were calculated by Eqs. (1 and 2) to develop productivity classification map for hilsa migration in the Bay of Bengal.

1 $$Gri{d}_{SWM}=({{\rm{Grid}}}_{{\rm{May}}}+{{\rm{Grid}}}_{{\rm{Jun}}}+{{\rm{Grid}}}_{{\rm{Jul}}}+{{\rm{Grid}}}_{{\rm{Aug}}}+{{\rm{Grid}}}_{{\rm{Sep}}}+{{\rm{Grid}}}_{{\rm{Oct}}})/6$$

2 $$Gri{d}_{NEM}=({{\rm{Grid}}}_{{\rm{Nov}}}+{{\rm{Grid}}}_{{\rm{Dec}}}+{{\rm{Grid}}}_{{\rm{Jan}}}+{{\rm{Grid}}}_{{\rm{Feb}}}+{{\rm{Grid}}}_{{\rm{Mar}}}+{{\rm{Grid}}}_{{\rm{Apr}}})/6$$

## Validation

Aqua MODIS results of spatial patterns of NPP in the Bay of Bengal were verified after field observation^2,6,52–61^. For this, a stratified simple random sampling was used in different habitats (e.g. estuary and delta, shelf water, and high sea) to identify 32 sites for subsequent assessment. Such an approach is appropriate to verify individual locations after the Aqua MODIS have been employed to assess spatial patterns of NPP in the Bay of Bengal. The productivity maps were verified by comparison between predicted productive zones and habitats of hilsa. For this purpose, geographical distribution map of hilsa by FAO, FishBase and GBIF (Global Biodiversity Information Facility), shads distribution by the Shad Foundation, and marine fishing zones of Bangladesh were used. In addition, participatory field visits, 120 semi-structured interviews and 60 focus group discussions were conducted with professional fishers in the coastal fishing villages of Cox’s Bazar, Chittagong, Noakhali, Laxmipur, Bhola and Patuakhali^51^. In addition, direct observations of hilsa in 55 landing centers were useful and meaningful way to confirm hilsa yields across space and time. The purpose of verification was to find out whether the existing hilsa habitats are in line with productivity classes or not. In addition, members of the academia, researcher and extension manager were consulted to verify the results of geo-spatial models and the distribution of productivity classes of this study.

## Results

### Net primary productivity (NPP)

**Figure 3:**
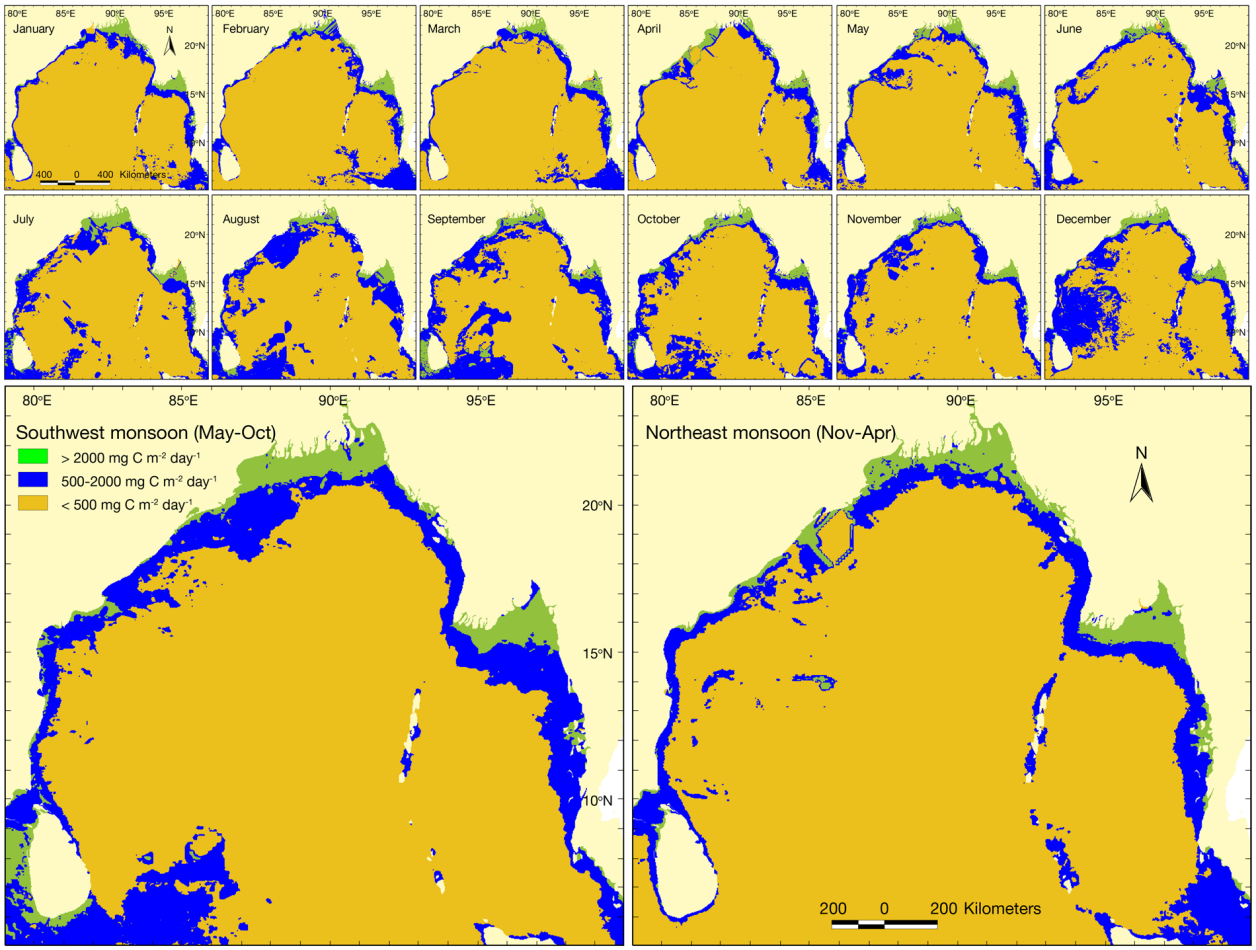
Monthly and seasonal variations of net primary productivity in the Bay of Bengal.

The most productive zone is designated with higher NPP and the productivity ranking is presented in Table 2. Monthly and seasonal NPP classification for the study area are illustrated in geo-spatial model (Fig. 3) and summarized in Table 3. After integration of respective months, 0.168 and 0.145 million km^2^ area were identified as the most productive zone with >2,000 mg C/m^2^/day during the southwest monsoon (May-Oct) and northeast monsoon (Nov-Apr) seasons respectively. There were 0.636 and 0.489 million km^2^ moderately productive zone with 500–2,000 mg C/m^2^/day, and 2.763 and 2.934 million km^2^ least productive zone with <500 mg C/m^2^/day (Table 3). The most productive zone was extended over 520 × 90–170 km (lying between 87–92°E and 21–23°N) in GBM delta, and 380 × 40–190 km (lying between 94–97°E and 15–17°N) in the Ayeyarwady delta. Moderately productive zone was confined to 1000 × 30–190 km in GBM delta and 980 × 70–180 km in the Ayeyarwady delta. While, 1540 × 40–130 km along the western boundary (i.e. Indian east coast) and 2130 × 50–180 km along the eastern boundary (i.e. Bangladesh-Myanmar-Thailand coast) of BoB basin were moderately productive. The offshore deep waters and high seas were the least productive zones.

**Table 2:** Ranking of different habitats according to net primary productivity.

Habitat type	General features	Ranking
Estuary and delta	GBM and the Ayeyarwady estuary and deltaic region	Most productive
Nearshore water	Nearshore and shallow waters (<200 m) within the exclusive economic zone (EEZ) of neighbouring countries along BoB	Most productive
Offshore water	Offshore deep waters (>200 m) within EEZ	Moderately productive
High seas	Open sea beyond EEZ	Least productive
Land, islands	Nearshore and offshore islands, and mainland	Constraints

**Table 3:** Productivity level and the corresponding area in the Bay of Bengal (total study area 3.568 million km^2^).

Month	Most productive		Moderately productive		Least productive	
	Area	%	Area	%	Area	%
January	0.149	4.17	0.379	10.62	3.040	85.21
February	0.133	3.74	0.407	11.41	3.027	84.85
March	0.131	3.66	0.404	11.32	3.033	85.02
April	0.159	4.46	0.373	10.46	3.035	85.08
May	0.156	4.39	0.378	10.59	3.033	85.02
June	0.136	3.81	0.478	13.40	2.953	82.79
July	0.213	5.97	0.592	16.59	2.762	77.43
August	0.149	4.19	0.803	22.51	2.615	73.30
September	0.190	5.33	0.861	24.13	2.517	70.55
October	0.166	4.65	0.707	19.81	2.695	75.53
November	0.139	3.90	0.508	14.25	2.920	81.84
December	0.156	4.38	0.861	24.13	2.551	71.49
SWM (May-Oct)	0.168	4.72	0.636	17.84	2.763	77.44
NEM (Nov-Apr)	0.145	4.05	0.489	13.70	2.934	82.25

SWM: southwest monsoon; NEM: northeast monsoon.

### Nutrients

**Figure 4:**
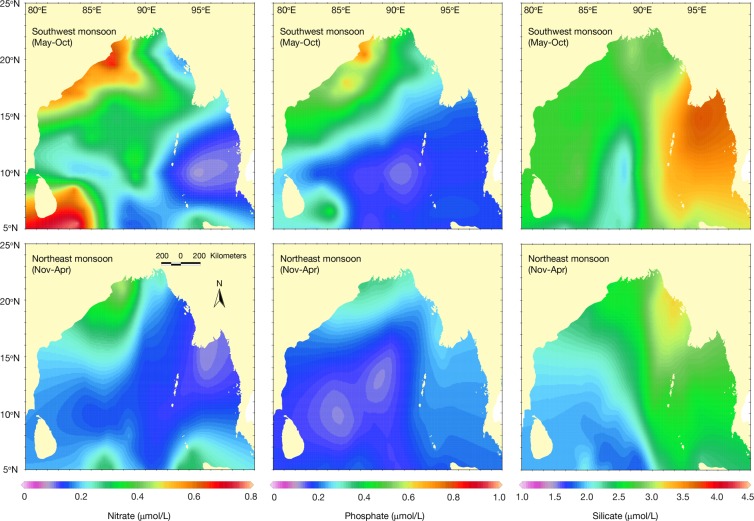
Spatial distribution of nutrient in the Bay of Bengal.

Geo-spatial distribution of nitrate in the upper 10 m indicated that northern and eastern BoB including GBM and the Ayeyarwady deltas were devoid of nitrate (<0.20 μmol/L) during northeast monsoon, but improved situation was evident with >0.60 μmol/L in western BoB along the Indian coast and Sri Lanka during southwest monsoon (Fig. 4). During northeast monsoon the western part of the GBM delta became enrich with nitrate (~0.40 μmol/L) than the east (>0.10 μmol/L), but the Ayeyarwady delta remained with minimum nitrate. The amount of phosphate was >0.4 μmol/L in GBM delta and in western BoB, while higher concentration of >0.6 μmol/L was recorded in the coastal waters (80–120 km wide) of eastern India during southwest monsoon. There were 0.2–0.3 μmol/L phosphate in GBM and Ayeyarwady deltas during northeast monsoon, while lower level (<0.2 μmol/L, a level same as the open ocean) observed in central and southern BoB. Irrespective of season, silicate distribution was higher (>2.5 μmol/L) in northern, western and eastern BoB and also in the deltas (i.e. GBM and Ayeyarwady) possibly due to freshwater influx and residual flow. Lower level of silicate (<2 μmol/L), which is typical for open ocean, was common in southern BoB.

### Zooplankton

**Figure 5:**
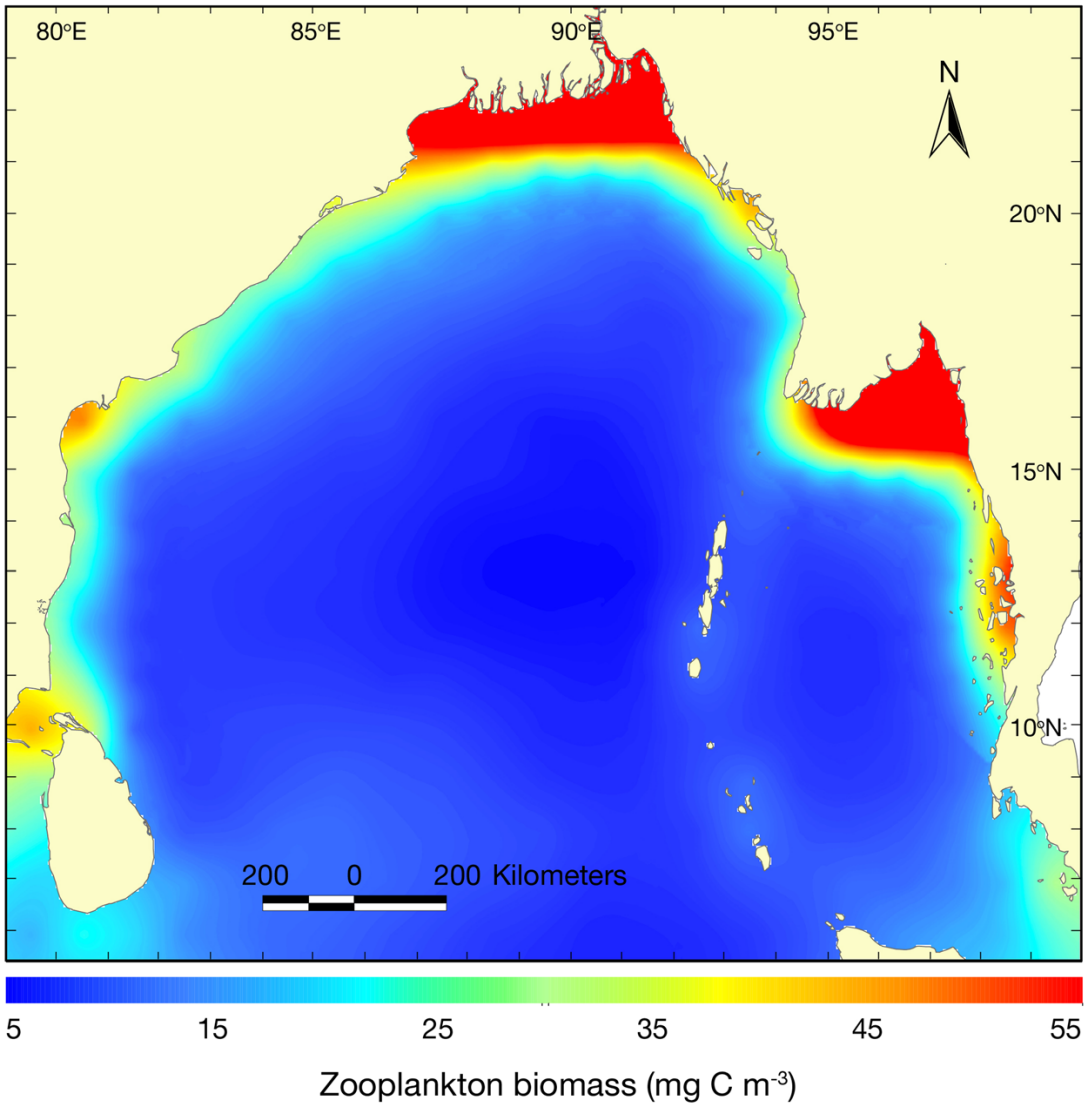
Spatial distribution of zooplankton biomass in the Bay of Bengal.

**Figure 6:**
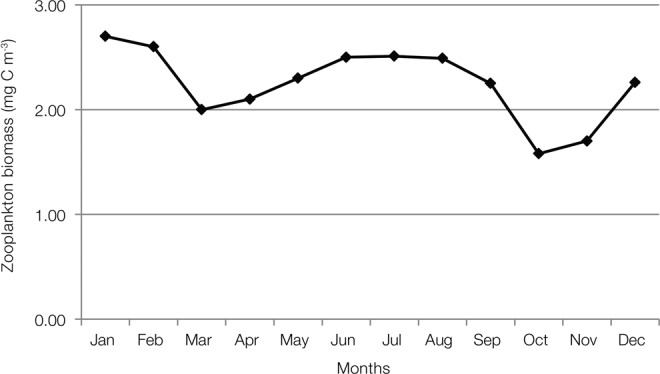
Temporal distribution of zooplankton biomass in the Bay of Bengal.

Spatial distribution of zooplankton biomass demonstrated that GBM and Ayeyarwady deltas had high zooplankton biomass (> 40 mgC/m^3^), while reduction of biomass was observed in the western BoB along the Indian coast and eastern BoB along the Myanmar coast (25–40 mgC/m^3^; Fig. 5). Lower zooplankton biomass (<10 mgC/m^3^) observed in central and southern BoB. However, the nearshore waters of Sri Lanka, Andaman-Nicobar and Sumatra indicated 15–25 mgC/m^3^. Spatial distribution of NPP and zooplankton biomass showed that NPP was significantly related with the zooplankton biomass (r = 0.65, p < 0.001). Temporal distribution of zooplankton biomass had minimum variation among the months, i.e. 2.60–2.70 mgC/m^3^ in January–February, followed by 2.49–2.51 mgC/m^3^ in June–August and 1.58–1.70 mgC/m^3^ in October–November (Fig. 6).

### Productivity and hilsa fishery

**Figure 7:**
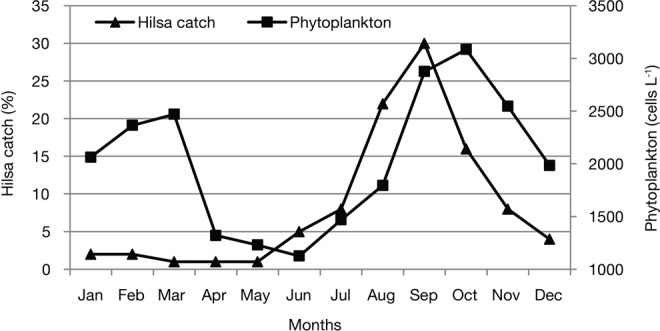
Phytoplankton abundance and hilsa yields (%) in the Ganges-Brahmaputra-Meghna (GBM) delta of the northern Bay of Bengal.

**Figure 8:**
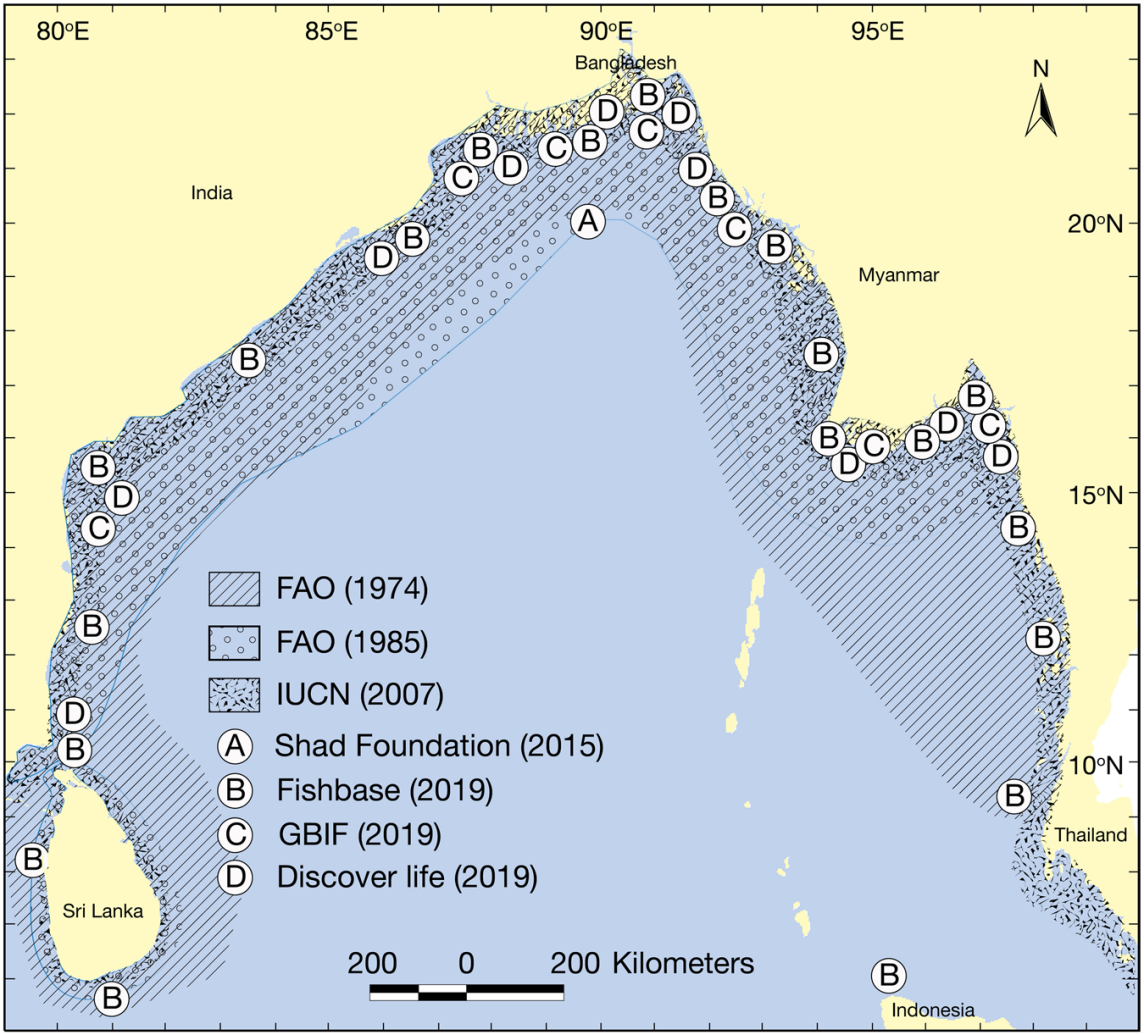
Spatial distribution of hilsa in the Bay of Bengal region since 1974.

Concurrent with the nutrient distribution, phytoplankton abundance in GBM delta had an enhanced concentration that also coincided with hilsa catch in the northern BoB at Bangladesh coast (Fig. 7). There were two peaks of phytoplankton in GBM delta of the northern BoB. The first peak of phytoplankton abundance with 3,085 cells/L was recorded in October, while the second peak with 2,470 cells/L was found in March. The peak period of plankton production in August-November was clearly linked to the highest hilsa catch (=~80% of 0.5 million tonnes annual catch), while the second peak in January-March can enhance growth and survival of hilsa juvenile that need scientific investigation. The model outputs for the productivity classes were accurately coincided with available maps of hilsa distribution from various sources such as FAO, FishBase, GBIF, IUCN, Shad Foundation and Discover life (Fig. 8). Acording to the fishermen, estuaries/deltas and nearshore waters are the most suitable zones for hilsa fishing. For that reason they operate hilsa gears within 80 m depth and a distance about 200 km from the coast. This fact suggests that suitable hilsa habitats are distributed in areas where primary productivity is typically high, varifying the model outputs for the productivity classes of this study.

Among 32 locations (i.e. 4 locations in the estuary and delta, 12 locations in the shelf water and 16 locations in the high sea) selected to verify the patterns of *in-situ* NPP distribution, the results of 27 locations were comparable to those Aqua MODIS data. Whereas, three locations in the estuary and delta (ID # 1, 2 and 4) and one location in the shelf water (ID # 15) were overestimated, and one location in the shelf water (ID # 9) was underestimated for NPP distribution. Thus, 84.4% of Aqua MODIS output did corroborate with field data (Table 4). The varification error matrix for Aqua MODIS imagery shows the incorrectly classified locations, based on 32 field verification sites (Table 5). MODIS accuracy (MA) and field accuracy (FA) for each of the ranking classes showed that high sea had the highest value of MA and FA (1.0). The shelf water was well discriminated from the rest of the classes (MA = 0.83 and FA = 0.83). The estuary and delta was poorly represented in the sampling (4/32) with MA and FA of 0.05 and 0.25, respectively. The Kappa Index of Agreement (KIA) was generated to determine the degree of agreement between the two outputs. Its value ranges from −1 to +1 after adjustment for chance agreement. A value of 1 indicates that the two outputs are in perfect agreement (no change has occurred), whereas if the two outputs are completely different from one another, then Kappa value is −1. The Kappa (K) and Kendall’s tau (T) coefficients had the value of 0.73 and 0.77 at 95% confidence, indicating that there is good agreement between field reference and Aqua MODIS data for NPP in the Bay of Bengal. We concluded that a high percentage of the pixels was classified correctly, better than would be expected by a completely random classification.

**Table 4:** Comparison of field observation data against Aqua MODIS ranking of NPP in the Bay of Bengal.

ID #	Habitat type	Latitude	Longitude	Field observationNPP (mgC/m^2^/day)	Aqua MODIS NPP ranking (mgC/m^2^/day)	Accuracy	
						ID #	%
1	Estuary and delta	20°24N	85°33E	1330	>2000	27	84.4
2	Estuary and delta	20°45N	89°55E	560	>2000		
3	Estuary and delta	12°25N	98°05E	2,590	>2000		
4	Estuary and delta	12°10N	98°30E	1920	>2000		
5	Shelf water	19°45N	86°10E	1080	500–2000		
6	Shelf water	17°25N	83°30E	1780	500–2000		
7	Shelf water	16°5N	82°50E	990	500–2000		
8	Shelf water	15°20N	80°45E	1750	500–2000		
9	Shelf water	10°55N	80°05E	2170	500–2000		
10	Shelf water	20°20N	87°36E	1190	500–2000		
11	Shelf water	19°31N	87°00E	1000	500–2000		
12	Shelf water	19°30N	87°38E	1000	500–2000		
13	Shelf water	20°10N	89°05E	820	500–2000		
14	Shelf water	17°20N	86°10E	300	500–2000		
15	Shelf water	16°25N	84°55E	310	500–2000		
16	Shelf water	10°30N	81°10E	690	500–2000		
17	High seas	20°N	88°E	190	<500		
18	High seas	18°N	88°E	180	<500		
19	High seas	15°N	88°E	350	<500		
20	High seas	12°N	88°E	320	<500		
21	High seas	15°35N	83°10E	189	<500		
22	High seas	17°01N	84°45E	220	<500		
23	High seas	18°05N	85°53E	214	<500		
24	High seas	18°10N	88°30E	73	<500		
25	High seas	15°30N	86°05E	200	<500		
26	High seas	20°N	88°E	427	<500		
27	High seas	18°N	88°E	155	<500		
28	High seas	15°N	88°E	202	<500		
29	High seas	12°N	88°E	216	<500		
30	High seas	9°N	88°E	204	<500		
31	High seas	15°N	88°E	336	<500		
32	High seas	20°N	88°E	184	<500

**Table 5:** Cross-tabulation for error matrix analysis of modeled Aqua MODIS data (row) against field reference data (column) for NPP in the Bay of Bengal.

	Field reference data				
	High sea	Shelf water	Estuary and delta	Row total	Field accuracy (FA)
**Aqua MODIS data**					
High sea	**16**	0	0	16	1.00
Shelf water	1	**10**	1	12	0.83
Estuary and delta	1	2	**1**	4	0.25
Column total	18	12	2	32	
MODIS accuracy (MA)	0.89	0.83	0.50		

Diagonal sum (bold) = 27; Overall Kappa = 0.73; Kendall’s tau = 0.77.

## Discussion

Geo-spatial distribution of nutrients in northern BoB is linked with runoff from GBM and the Ayeyarwady river systems signifying an upward pumping of nutrients from subsurface zone that overlaps with Madhupratap *et al*.^6^. Muraleedharan *et al*.^52^ recorded <0.1 μM nitrate, 0.4 μM phosphate and >4 μM silicate in the open waters of BoB which is almost similar to present findings. In contrast, Choudhury and Pal^53^ recorded 11.13–24.19 μM nitrate, 2.23–9.41 μM phosphate and 19.97–127.32 μM silicate along the southeastern coast of India, higher than the present study. Rao *et al*.^62^ and De Sousa *et al*.^63^ mentioned that rivers flowing into BoB might not contribute much to the inorganic nutrient pool as substantial part of the terrigenous materials are lost at its confluence due to oceanographic processes^64^. However, river discharges are associated with greater lithogenic fluxes in BoB^65^, where biogenic matters may rapidly scavenged along with terrigeneous origin and ballasts the materials in faster sedimentation to the deeper ocean^66,67^. Rivers and atmosphere can supply 20% nitrogenous inputs to BoB, while 80% nitrate comes to the surface from deeper waters by the cyclones, tidal surges, depressions or high speed winds occurring frequently in BoB during Oct-Nov and Mar-Apr^68^. In general, GBM and the Ayeyarwady deltas are categorized as eutrophic as levels of inorganic nutrients remain high throughout the year compared to the oligotrophic reference values (e.g. phosphate 0.011–0.077 µM, nitrate 0.087–1.900 µM, primary productivity 0.135–0.143 mg Cm^−3^ h^−1^) of Karydis^69^ and Ignatiades^70^. Continental water flow, nutrients and organic matters originated from the upstream rivers maintain ecosystem functions in the deltas and supply food to the resident species including hilsa^71^. For instance, the crisscrossed rivers/tributaries of the GBM and Ayeyarwady deltas have an intimate relationship with surrounding land-based activities, such as agriculture, forest, wet meadow, human settlement, industrial development, port operation and tourism activities that play important roles on aquatic habitats. Some areas, such as the offshore deep waters and high seas, are not known as suitable hilsa habitats, but the reasons behind the fact need to uncover with scientific interpretation. Moreover, geo-spatial models can assess suitable habitats of hilsa across the life cycle^51^, which requires data on bathymetry, oceanographic processes, water quality, primary productivity and habitat characteristics specific to life stages. Some other methods including single nucleotide polymorphism technology (SNP), ^87^Sr/^86^Sr isotope ratios in otoliths^72^, allozymes and morphometric analysis^73^, and distinctive trait of the parasite fauna^74^, can provide information about the seasonal movements and residency of hilsa.

**Figure 9:**
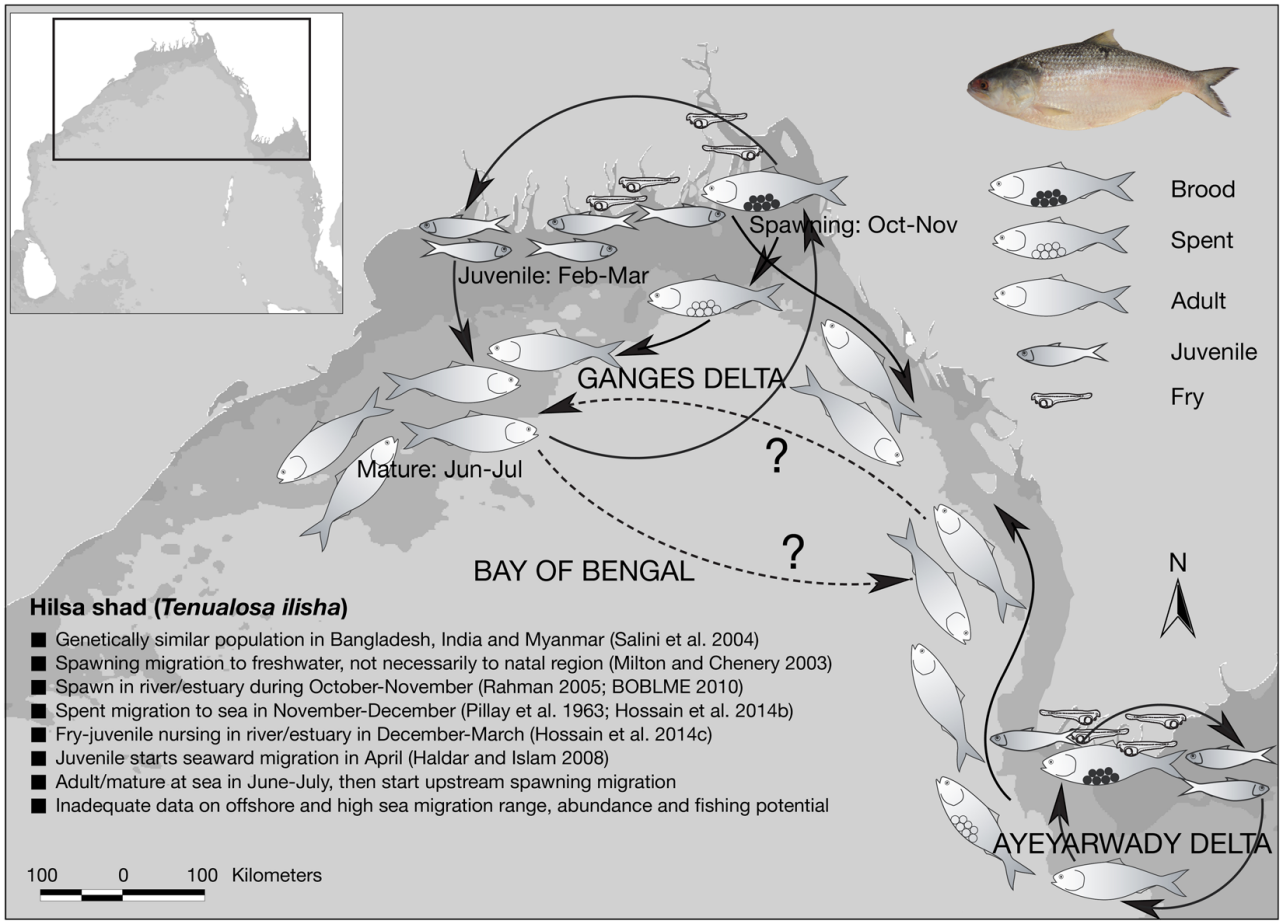
Conceptual map of hilsa habitats, their migration pattern and life cycle in the northern Bay of Bengal.

**Figure 10:**
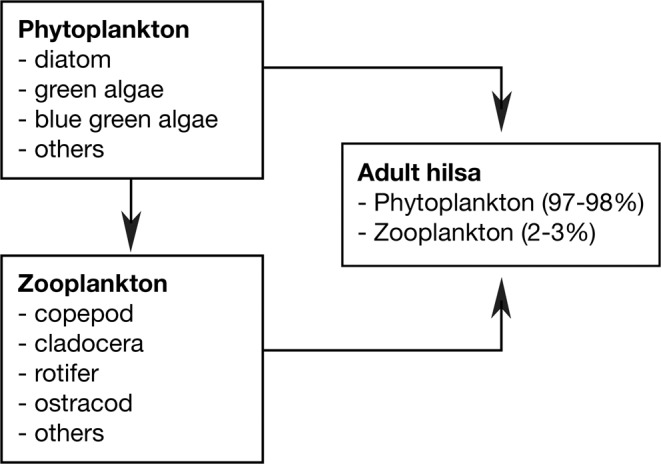
The feeding interactions of hilsa shad.

High productivity in the nearshore and deltaic waters of BoB integrates with high ambient nutrients. The distribution of NPP positively correlated with phytoplankton concentration (r = 0.75, p < 0.001) that implies that pelagic-neritic fishes like hilsa is expected to maintain their positions within productive areas for sustaining their growth and maturity. Hilsa fed on algae, diatoms, copepods, cladocerans, protozoa, rotifers and the larvae of molluscs^24,75^. The relationship between hilsa and the abundance of their diets suggests that habitat of hilsa is restricted up to the mixed layer depth (e.g. 20–40 m), which is possibly above the thermocline having maximum level of NPP. At this depth, phytoplankton, copepods, cladocerans and protozoa may be more intense^76^ and more competently obtained. The present study indicate two peaks of phytoplankton in August-November and in January-March with the lowest abundance in April-July, and this data corresponded well with Choudhury and Pal^53^ who reported maximum phytoplankton (1,611 cells/mL) in December and minimum (494 cells/mL) in July along the eastern Indian coast of the Bay of Bengal. Incidentally, spawning grounds of hilsa are located in GBM delta^77^ and Ayeyarwaddy delta^78,79^, suggesting that these productive water bodies are suitable for hilsa to retain the larvae and juveniles, as illustrated in Fig. 9. In this connection, hilsa migrates to GBM delta for spawning in September-October^77^ when the levels of NPP and abundance of phytoplankton are high in the system. In addition, coastal rivers and nearshore shallow waters in GBM deltaic region are also suitable nursery grounds for hilsa juveniles until March^80^. April onwards juveniles start seaward migration as the second phase of anadromous behavior and moves up to 250 km from the coast^81^ with daily travels of about 71 km^82^. Moreover, Day^83^ and Milton^84^ mentioned that hilsa spends part of life in the sea but not far from the shallow coastal belt. However, operational limitations of gears made difficulties to determine the abundance, extent of seaward migration and fishing potential of hilsa in offshore and high seas due to lack of data and observations. Consequently, Hossain *et al*.^33^ recommend for comprehensive study in determining the range of hilsa migration with spatial and temporal distribution across the life cycle. It is evident from the present study that being plankton feeder, hilsa tend to follow plankton rich areas and keep moving from one place to another in search of productive zones and continue to grow. For example, sardine larvae requires 5.7–9.6 mg C/day of primary productivity^85^ while maximum consumption rates for 1, 2 and 3 years old sardine are 0.042, 0.012 and 0.0049 g-prey g-fish^−1^ day^−1^, respectively^22^. Thus, nursery ground of sardine larvae is located in high productivity zone, where adult sardine (1–3 years old) can live in relatively low productive zone, similar information are not available for hilsa that need to examine. Hilsa prefers zooplankton in early stages and shift towards phytoplankton in adult stage^86,87^. Diatom, green algae, and blue green algae represent phytoplankton menu of hilsa, while zooplankton menu comprises copepod, cladocera, rotifer, and ostracod^23,88–91^. Adult hilsa comprised 97–98% phytoplankton with only 2–3% zooplankton^24,92,93^ (Fig. 10).

The relationship between hilsa yield and NPP could be interesting to forecast how hilsa population in BoB might respond to future changes in productivity. The *Galathea* Expedition in 1950–1952^54^ measured NPP 0.1–0.3 mg C m^−2^ d^−1^ in the deep sea and 0.01–2.16 g C m^−2^ d^−1^ in the shelf region of BoB^6^. Data of the subsequent studies from different regions of BoB are given in Table 6. Thaw *et al*.^55^ proposed 300–500 g C m^−2^ year^−1^ as standard reference value of NPP for eutrophic region. The present study found NPP of >2,000 mg C m^−2^ day^−1^ in GBM and the Ayeyarwady deltas that coincided with Thaw *et al*.^55^, i.e. 2,590 ± 1,569 mg C m^−2^ day^−1^ at the Ayeyarwady delta. Conversely, a drop in primary productivity ranging 500–2,000 mg C m^−2^ day^−1^ was noted in the area below 22°N along the western and eastern boundary of BoB basin. The least productivity of <500 mg C m^−2^ day^−1^ was found in deeper part of BoB and the Andaman Sea. Though no specific trend was observed in seasonal variations, August-October represented with higher productivity in GBM delta, and lower productivity found in April-June that agrees with the findings of Routray and Patra^94^, Mohanty *et al*.^13^, Kumar *et al*.^2^ and Radhakrishna *et al*.^95^. Phytoplankton production also follows similar pattern, i.e. phytoplankton abundance is high during August-October in GBM delta. Thus, deltas, estuaries and shelf regions of the northern BoB have higher NPP that may support rich neritic and pelagic fisheries. For instance, Sarmiento *et al*.^95^ used coupled climate models to forecast responses of NPP in 2040–2060 and predicted that NPP in the sub-polar North Pacific could increase by 10–20% that could result 20% increase in carrying capacity of pelagic species, such as herring. In this context, distribution of NPP and nutrients in the northern BoB including mega deltas and shelf regions can explain and predict the distribution of pelagic fishes such as hilsa fishery in Bangladesh.

**Table 6:** Reported net primary productivity (NPP) from different areas/regions of the Bay of Bengal.

Area/region	Season	NPP (mg C m^−2^ d^−1^)	Reference
GBM delta	Southwest and northeast monsoon	>2,000	Present study
Ayeyarwady delta	Southwest and northeast monsoon	>2,000	Present study
Nearshore water	Southwest and northeast monsoon	500–2,000	Present study
Offshore water	Southwest and northeast monsoon	<500	Present study
High sea	Southwest and northeast monsoon	<500	Present study
Ayeyarwady delta	Dry season (Apr–May)	2,590 ± 1,569	Thaw *et al*.^55^
Ayeyarwady delta	Rainy season (Aug–Sep)	1,700 ± 1,100	Thaw *et al*.^55^
GBM delta	March–April	1330	Routray and Patra^94^
Northern BoB	Summer	433.8	Kumar *et al*.^2^
Southern BoB	Summer	502.01	Kumar *et al*.^2^
Coastal waters of the western BoB	December	788.88	Choudhury and Pal^53^
Coastal waters of the western BoB	July	44.44	Choudhury and Pal^53^
Open ocean of BoB	July–August 2003	73–200	Muraleedharan *et al*.^52^
Coastal waters of the western BoB	July–August 2003	108–357	Muraleedharan *et al*.^52^
Open ocean of the BoB	April–May 2003	155–427	Prasanna Kumar *et al*.^112^
Offshore water of the western BoB	April–May 2003	250–469	Prasanna Kumar *et al*.^112^
Coastal waters of the western BoB	Summer	350 ± 225	Madhu *et al*.^58^
Coastal waters of the western BoB	Winter	252 ± 210	Madhu *et al*.^58^
BoB	September–October 2002	90–870	Kumar *et al*.^68^
BoB	April–May 2003	154–975	Kumar *et al*.^68^
Shelf water	Southwest monsoon	39.7–502.0	Madhupratap *et al*.^6^
Offshore water	Southwest monsoon	89.4–220.7	Madhupratap *et al*.^6^
Open ocean of BoB	September–October 2002	184–512	Gauns *et al*.^113^
Coastal waters of the western BoB	Pre-southwest (March–April)	1050	Gomes *et al*.^114^
Open ocean of BoB	Pre-southwest (March–April)	160	Gomes *et al*.^114^
Offshore water	August–September 1978	180–2200	Bhattathiri *et al*.^115^
Western BoB	August–September 1978	120–3410	Bhattathiri *et al*.^115^
Offshore water	August–September 1976	129.99–329.45	Radhakrishna *et al*. (1978)^61^
GBM delta	August–September 1976	49.66–606.37	Radhakrishna *et al*.^61^
High sea	Galathea Expedition 1951	0.1–0.3	Brunn *et al*.^54^
Shelf water	Galathea Expedition 1951	0.01–2.16	Brunn *et al*.^54^

**Figure 11:**
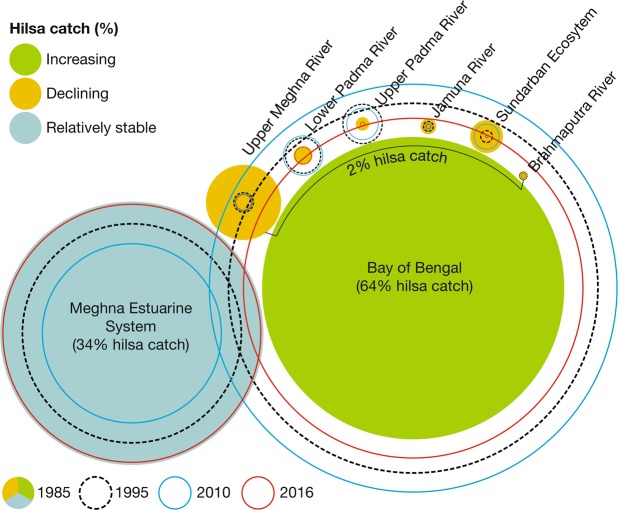
Spatial variations in catch data explain the habitat and movement routes of hilsa in the Ganges-Brahmaputra-Meghna (GBM) delta (data source: Department of Fisheries, Bangladesh).

Data suggests that total hilsa catch in Bangladesh exceeds nine million tonnes since 1983–84. Specifically, hilsa catch has increased to 517,198 tonnes in 2017–18 from 146,082 tonnes in 1983–84, an annual growth of 7% in the past three decades. The marine waters of northern BoB share the major catch (65%), where the remaining portion fished from the Meghna estuarine system (33%) and from several rivers/tributaries (2%). As the instance in GBM delta, spatial variations in hilsa catch (Fig. 11) interpret habitat suitability and movement routes that can enhance conservation and management initiatives of hilsa fishery. The geographical distribution of hilsa along the coast of Bay of Bengal has been documented by FAO (Fischer and Whitehead 1974^96^; Whitehead^97^), IUCN^98^, shad foundation^99^, FishBase^100^, Global Biodiversity Information Facility (GBIF)^101^ and Discover life^102^ (Fig. 8). Moreover, their occurrences in the adjacent rivers were reported by Hora^103^, Motwani *et al*.^104^ and Quereshi^105^. Interestingly, hilsa distribution maps with modelled year 2100 native range map based on IPCC A2 emissions scenario (Aquamaps)^106^ has endorsed similar range for hilsa. Indeed, major efforts of hilsa fishing in Bangladesh have been concentrated within about 100 km from the coast^107^.

The majority of global oceanic data have been collected through cruises for specific periods which is expensive and not many research institutions and country can afford, especially the developing countries like Bangladesh. As a proxy, satellite imagery is useful for specific temporal resolution of any area during the routine observation schedule. For example, MODIS captures data in 36 spectral bands (0.4–14.4 μm wavelength) for 250–1000 m spatial resolutions with viewing swath of 2330 km and the revisit cycle one to two days. MODIS utilizes four on-board calibrators to provide in-flight calibration, whereas vicarious calibration enhances by using marine optical buoy. Thus, the shortwave infrared (SWIR) bands enable more robust atmospheric corrections in turbid coastal waters (Wang *et al*.)^108^, while the band around 685 nm is important for detection of phytoplankton fluorescence^109,110^ (Gower *et al*., 1999; Hu *et al*., 2005). Thus, a comparison among the observation/cruises can enhance scientific understanding and interpret the validation process that we applied in this study. Nevertheless, primary productivity is important for pelagic fisheries recruitment, growth and yield, and found to largely controlled by variation in seasonal temperature gradient^56^, freshwater plume and haline stratification^40,43,44,57^, vertical transfer of nutrients from the subsurface levels into the euphotic zone^111^ and dissolved oxygen, which were out of scope of this study. Therefore, further studies are necessary to investigate the multicriteria evaluation of above mentioned parameters to clarify the relationships of hilsa and its habitat conditions.
